# Formation mechanism of contributors’ self-identity based on social identity in online knowledge communities

**DOI:** 10.3389/fpsyg.2022.1046525

**Published:** 2022-12-15

**Authors:** Tongfei Gu, Zhichao Cheng, Zeqian Zhang, Cui Li, Yuan Ni, Xiaokang Wang

**Affiliations:** ^1^School of Economics and Management, Beihang University, Beijing, China; ^2^Institute of Disaster Prevention, Hebei, China; ^3^School of Economics and Management, Beijing Information Science and Technology University, Beijing, China; ^4^School of Economics and Management, Beijing University of Posts and Telecommunications, Beijing, China

**Keywords:** online knowledge community, social identity, self-identity, information support, novel posting, sense of self-worth, sustainable development

## Abstract

**Introduction:**

Contributors’ self-identity is a critical element in the sustainable development of online knowledge communities (OKCs). However, research concerning the formation mechanism of contributors’ self-identity remains scarce. This research posits information support, novel posting and sense of self-worth as mediating variables between social identity and self-identity to construct a path model, aiming to explore the way in which self-identity is formed on the basis of social identity in OKCs.

**Methods:**

To examine this mode, an online survey was administered to many different OKCs, and 515 usable questionnaire responses were collected. Structural equation modeling was then employed to examine the model.

**Results:**

The R2 value of self-identity was 0.627, thus indicating that the model was able to explain 62.7% of the variance in self-identity. We find that self-identity emerges through the mediating effects of information support, novel posting and sense of self-worth. In addition, social identity can elicit novel posting and information support, which are all beneficial for enhancing the dynamics of OKCs and further generate sense of self-worth. We also observe that although social identity and individualized behavior (novel posting) are generally incompatible, they can be compatible in the context of OKCs.

**Discussion:**

Self-identity as a contributor can be formed on the basis of social identity via the social path and the psychological path in OKCs, while the two paths for fostering self-identity are not independent of each other, and there is also a very strong link between behavioral and psychological mechanisms.

## Introduction

In recent years, given the increasing popularity of mobile internet use, people’s access to the internet has become more convenient, and the amount of time people spend online has increased significantly (Zhao and Shi, 2022). Accordingly, people’s life scenes have started to shift from offline to online, virtual communities have emerged and developed rapidly, and virtual community engagement has become a significant and essential factor in people’s daily lives (Chou et al., 2010; Huangfu et al., 2022). Virtual communities are internet-mediated social gatherings based on shared values, purposes, and interests (Shen et al., 2010), where users can openly exchange information and even experience personal emotions and develop interpersonal relationships. An online knowledge community (OKC) is a kind of virtual community that serves as a network platform for the primary purpose of sharing knowledge. On this platform, users with common interest in a certain field of knowledge congregate and exchange knowledge and experiences through the internet to achieve knowledge acquisition, dissemination and innovation (Fang and Zhang, 2019). In the 21st century, human society has entered the era of the knowledge economy, and knowledge has become an extremely important factor in contemporary production (Kim et al., 2022). With the rise of Web 2.0, technology has given users more active rights, so they can function both as consumers and creators of information content. The emergence of OKCs has fully mobilized the knowledge surplus of the whole society, provided an effective platform and tool for users to manage knowledge, facilitated the dissemination of knowledge among different users, and promoted users’ knowledge replenishment and knowledge advancement (Guan et al., 2018).

Although virtual communities are not hierarchical organizations and do not feature hierarchical relationships among group members, some studies have indicated differences in status among members (Mutter and Kundisch, 2014). Tsiotakis and Jimoyiannis (2016) divided members in virtual communities into three categories, namely, contributors, common members, and lurkers. Contributors make the most important contributions to the virtual community by posting high-quality content (also known as novel posting) and actively answering other members’ questions (also known as information support), and they therefore have a high reputation and good credibility and constitute the most influential of the three categories of members. Through empirical research, Valente and Pumpuang (2007) found that contributors play a significant role with respect to shaping public opinion in virtual communities because they can influence the attitudes and behaviors of other users through their status, leadership and expert capability. Lurkers are silent participants in virtual communities, who often merely browse information without posting or participating in discussions (Tsai and Kang, 2019). As such, lurkers are the least influential of the three categories of members. From the perspective of content contribution, common members rarely contribute high-quality content to the virtual community and are more likely to evaluate or discuss the posts of contributors, so their level of influence lies between those of contributors and lurkers (Li et al., 2013). The roles of members in a virtual community are not prespecified but are determined by the behavior of the members. Once members are aware of their role in the community, their behavior becomes role aligned (Xiong et al., 2018). Self-identity, also known as role identity, refers to the perception and internalization of one’s role identity (Rousseau, 1998). Some studies have indicated that individuals come to understand their identities by placing themselves in a social environment (Rousseau, 1998; Liu and Yang, 2022), and their behavioral performance can be a measure of identity (De Cremer and Oosterwegel, 2000); therefore, engaging in contribution behaviors helps people perceive their self-identity. A higher level of self-identity increases both people’s motivation to play the role in question and their likelihood of making efforts to play the role. Therefore, the formation of self-identity as a contributor foreshadows the establishment of his or her role as a contributor (Zhang and Bartol, 2010). There has been a paucity of research on how self-identity is formed. The little research that exists only suggests that social identity can generate self-identity as a contributor, but it is not clear how it is formed based on social identity. To fill this theoretical gap, this research uses a dual self-running perspective to analyze the formation and reinforcement mechanisms of self-identity as a contributor based on social identity.

In traditional enterprises, a workgroup is a kind of “heter-organization” because it is established and guided by external commands. An OKC, in contrast, is a “self-organization”; that is, the community relies on external instructions but rather on its own orderly operation to facilitate community functions. There are two self-running mechanisms in OKCs: the self-running mechanism between social identity and contribution behavior and the self-running mechanism between self-identity and contribution behavior. Thanks to these two self-running mechanisms, OKC has been able to prosper and grow.

The self-running mechanism between social identity and contribution behavior is described below. Social identity reflects members’ psychological, emotional, and interpersonal attachments to the group and their sense of belonging to the group (Bagozzi and Dholakia, 2002; Farivar and Wang, 2022). Social identity can enhance members’ sense of integration into the group and recognition of the group’s values, thereby allowing them to engage in more contribution behaviors and helping the group achieve its goals (Wang et al., 2022). Hsu et al. (2012) defined the behavior of virtual community members who actively contribute their time and energy to benefit or help other members as contribution behavior. Contribution behavior is significantly different from simple community use behavior, as the former reflects the individual’s willingness to provide value to others (He et al., 2012). The goal of joining an OKC is to be able to acquire relevant information quickly and accurately, which occurs in two ways: first, by searching the posted content in the community to obtain high-quality information and second, by taking the initiative to ask one’s own questions and receiving solutions to problems through other members’ replies. Novel posting and information support are typical contribution behaviors, as both fully satisfy the purpose of members’ participation in OKCs. It is costly for members to make novel posts and receive information support, since these behaviors require not only their professionalism but also a great deal of time and effort; accordingly, only group members with a strong sense of social identity are likely to engage in these two contributing activities. Members who perform contribution behaviors (novel posting and information support) are important to OKCs, and because these members make important contributions to the community, they can receive a great deal of social prestige, thereby enhancing their self-esteem. Numerous studies have found that a member’s high importance to the group generates high self-esteem (Hsu et al., 2012; Miljeteig and von Soest, 2022), and because members prefer to belong to groups that can provide them with high self-esteem, group members that are highly important to a group will exhibit high levels of social identity with respect to OKCs (Simon et al., 2016; Hong et al., 2021). Based on these conclusions, this self-running mechanism of OKCs manifests when social identity generates novel posting and information support, which can conversely facilitate members’ social identity.

The self-running mechanism between contribution behavior and self-identity is described below. At the behavioral level, members can perceive their roles as contributors by engaging in novel posting or information support behaviors; at the psychological level, members performing these two contribution behaviors generate a sense of self-worth, which causes members to clarify their roles as contributors and further improves self-identity (Schlenker, 1975; Yang et al., 2022). Once an individual identifies with his or her role as a contributor, he or she can continue to contribute to the community and to perform contribution behaviors (Simon et al., 2016). In summary, novel posting and information support can generate self-identity directly through the social path or employ the mediating effect of sense of self-worth to cultivate self-identity through the psychological path. In turn, self-identity promotes novel posting and information support.

As a kind of self-organization, the orderly operation of OKCs relies on a dual self-running mechanism. In this context, social identity is the point of origin for this dual self-running mechanism, contribution behavior (novel posting and information support) is its core mediating variable, and self-identity is an important outcome.

Both self-identity and social identity are a form of identity building, but there are significant differences between them. Self-identity is concerned with a specific role within a group, while social identity is concerned with an identity of membership embedded in a group, but does not refer to a specific role assumed within that group. All three types of members (lurkers, common members and contributors) can have a social identity in OKCs, but only contributors will have self-identity as a contributor in OKCs. Previous studies have not fully identified the formation process of contributors’ self-identity or addressed how and which variables constitute the formation pathway. We constructed and empirically demonstrated a path model to identify the process of forming contributors’ self-identity in OKCs. Contributors’ self-identity can emerge from social identity *via* the mediating effects of the relevant variables (information support, novel postings, and sense of self-worth).Our research may also provide operators of OKCs with theoretical and practical insight into achieving sustainable operations.

## Theoretical background

### Social identity

Social identity refers to “individuals’ self-concept in light of their group membership, which usually has significant value and emotional significance” (Tajfel, 1982). From the perspective of social psychology, social identity is divided into three levels that exhibit a progressive relationship (Ellemers et al., 1999). Value identity characterizes the positive or negative evaluations that individuals make of their own values based on their group membership, emotional identity reflects the psychological satisfaction that an individual obtains from belonging to a group and the positive feelings of that individual toward group members, and cognitive identity reflects the individual’s ability to recognize his or her similarities with other members of the same group and differences from members of other groups (Postmes et al., 2005). Individuals tend to belong to groups that can improve their sense of self-worth, which allows individuals to develop a good sense of self; in turn, these improvements promote a sense of integration and emotional connection to the group. Therefore, value identity is positively related to emotional identity. In virtual communities, individuals’ perception of similarity among members is expressed concretely by the belief that group members have common goals, interests, and values. When individuals develop stronger positive emotions toward the group, the status of the group in the individual’s mind is improved, resulting in a stronger connection between group characteristics or images and the individual’s self-concept (Easterbrook and Vignoles, 2013; Weber and Maffezzolli, 2021). Due to the anonymity and temporal separation of virtual communities, individuals’ perceptions of other members are often based on a vague impression; that is, individuals’ perceptions of other members are often based on an overall impression of the group (Ren et al., 2007). Therefore, emotional identity positively affects cognitive identity.

The process of establishing social identity is divided into three stages: social categorization, social comparison, and positive distinction (Turner, 1975). First, through a process of social categorization, individuals assign themselves to a group and perceive themselves as having the universal characteristics shared by in-group members; subsequently, individuals make a social comparison of the group they have joined with other groups in terms of a particular characteristic, specifically comparing strengths and weaknesses; finally, individuals cognitively exaggerate similarities with the in-group and differences from other groups, evaluating in-group members more positively while evaluating out-group members more negatively, thus completing the positive distinction process (Ashforth and Mael, 1989).

Social identity theory explains that an individual’s perception of group membership is an important determinant of his or her thoughts and behaviors, and it suggests that social identity is a bridge between the individual and the group at both the psychological and behavioral levels (Bhattacharya and Sen, 2003; Walker, 2021).

### Self-identity

There is no consensus concerning the concept of role in academic circles. One definition of role emerges from the sociological conception of social role and focuses on the perspective of social relations, social norms, social status, and social identity (Hinkle et al., 1989). For example, a role entails acting in accordance with the norms specified by the social structure, and each role contains a set of rights and obligations as well as a system of behavioral norms. Another definition of role is the social psychological view of social roles, which focuses on the perspective of individual behavior and behavior patterns. For example, a role entails a pattern of behavior associated with a certain social position and refers to the behavior expected of a person who occupies a certain social position (Hogg and Hardie, 1991; Wheeler and Bechler, 2021). Role refers to a set of behavioral patterns in which an individual occupies a particular social position in a social relationship and through which his or her behavior conforms to social expectations. In other words, roles are a combination of an individual’s specific status as determined by certain social relationships, society’s expectations of the individual, and the behavioral patterns displayed by the individual (Hewitt et al., 2003).

Self-identity refers to the perception and internalization of one’s role identity (De Bruijn et al., 2012). Role expectations and meanings constitute a set of guidelines for individuals’ behavior. Therefore, an individual’s identification with a self-role identity often represents that part of his or her self-concept that is closely tied to a particular behavior, which becomes an important part of the individual’s self-concept (Hagger and Chatzisarantis, 2006; Spinelli, 2021). Therefore, self-identity can predict individual behavior. Key to identity theory is the establishment of a causal link between role identification and behavioral intentions (Hustvedt and Dickson, 2009). Individuals generally categorize themselves into specific, explicit social roles that guide their goals and practices to the extent that they spontaneously act in ways that correspond to the roles with which they identify (Sparks and Shepherd, 1992; Mamman et al., 2022).

In virtual communities, member roles are not predesignated but are determined by members’ behavior, and so once members are aware of and identify with their roles in the community, their behavior matches the identified roles (Chiregi and Navimipour, 2016). Contributors, common members, and lurkers play their respective roles in the processes of content generation, evaluation, and dissemination in the context of virtual communities, and so each of these roles constitutes an indispensable part of the process of the orderly and healthy operation of virtual communities, although contributors play the most prominent role and make the greatest contribution (Wu and Chiang, 2021).

### Contributors

In the free and open environment of online knowledge communities, a small number of members make great contributions to the community *via* their novel posting and information support behaviors, thus gaining social prestige and recognition from other group members; in contrast, other members focus on reading their postings when browsing the information in the community (Wasko et al., 2009; Kiselev et al., 2022). From the perspective of communication science, this small group of the most influential members in the community can be called contributors.

There are two types of contributors in OKCs: professional contributors and active contributors. Professional contributors are those who have deep knowledge of the common interests of the community, possess excellent professional skills and are willing to impart professional knowledge and experience to other members of the community or to provide unique insights into issues raised by others (Turcotte et al., 2015). Due to the virtual nature of cyberspace and the anonymity of netizens’ identities, there is a lack of trust when members communicate with each other; therefore, people tend to judge the professionalism of posters by the quality of their posts, and posting content that includes detailed descriptions, profound profiles or unique insights is often recognized by others. Accordingly, posters who make novel posts are seen by other members as having expert capability. Professional contributors have the greatest advantage of being proficient in a particular area of common interest; they do not necessarily post often, but the quality of their posted content is high. Active contributors are those who become centers of attention in the community by posting and replying to posts frequently and by posting new knowledge and information in areas of common interest in a timely manner (Spears, 2021). They are willing to share their knowledge with others and actively answer others’ questions, thus gaining recognition from other members and high social prestige.

In an empirical study of virtual communities, Wasko et al. (2009) found that a mere 4% of members contribute nearly 50% of the community’s content, and these authors referred to such members as contributors. This finding shows that although contributors are a small percentage of the population of the community, they make an enormous difference.

## Research model and hypotheses

### Social identity, novel posting, and information support

Novelty posting refers to posts whose content is creative and unique (Sethi et al., 2001). Novel posts in OKCs are characterized by two features: professionalism and differentiation (Carmel et al., 2012). Professional is the core of the post, while differentiation is its outward expression. These two characteristics are directly related, and it is precisely the characteristic of professionalism that leads these posts to be distinguished from ordinary posts. The degree of novelty mirrors the quality of the content posted and is therefore the most reasonable indicator of content quality (Brake, 2014). Novel posting enriches the content in the OKC and improves the interactivity and vitality of the OKC by attracting a great deal of attention and many replies. Novel posting often leads to lively discussions, in which the interaction process is enjoyed or engaged by initiators, participants and viewers, resulting in an intense and positive community experience. Obviously, novel posting has a critical impact on the successful establishment and development of a thriving OKC. In OKCs, social identity contributes to community cohesion and the internalization of community norms by individual members; it contributes to individual members’ effort, engagement, job satisfaction, organizational citizenship behavior, and job performance, which in turn contributes to the overall benefits of the community. At the same time, a high level of social identity contributes to the tolerance of different cultures and good interpersonal relations, which in turn helps to stimulate individual creativity (Khan, 2021). Members of a virtual community can provide help to other members by posting comments or replies in a variety of ways, such as through text, voice and pictures, an act known as social support (Rotman and Preece, 2010). In virtual communities, social support is divided into two dimensions: information support and emotional support (Hajli et al., 2015). As functional value is the most important value of OKCs, social support in OKCs mainly refers to information support, i.e., providing targeted methods, advice and resources to help other members solve the practical problems they encounter (Zhou and Li, 2014). When members encounter new knowledge they do not understand or new problems they cannot solve on their own, participating in OKCs is a more effective way of accessing information or solving problems. As information support can be targeted at the problems of group members, it is a typical act of community contribution. In OKCs, social identity enhances member engagement, which refers to a long-term, values-driven relationship formed between members and the virtual community that fosters interaction between individual members and others, enhances individual members’ emotional or behavioral commitment to the virtual community, and is the underlying internal motivation for members to help or cooperate with each other (Han et al., 2022). The member’s engagement behavior is often active, voluntary and not part of the member’s responsibility in the economic transaction activity (Busalim et al., 2021). If members have a high level of cognitive, emotional and behavior engagement with the virtual community, they will show strong satisfaction, trust, loyalty and commitment to the virtual community, and will therefore actively interact and cooperate with others (Elsayed, 2021), thus creating good experiences and values with others. Thus, social identity helps members develop altruistic behavior.

Members’ social identity reinforces the need for members to maintain their particular identity and to internalize the group’s values as a personal code of conduct (Mael and Tetrick, 1992), thus leading to a preference for the in-group and consequently more contribution behavior (e.g., novel posting and information support).

Individual members all have social and belonging needs, and novel postings are a form of self-presentation for posters, who also want their postings to attract the attention of others (Jacobsen et al., 2017). Novelty is a valid indicator of the quality of the posting content and determines how attractive the posting content is to the viewer, as well as the attractiveness of the virtual community (Carmel et al., 2012). Novel posting is more likely to attract attention and even praise from others as well as to inspire more replies (e.g., through other members asking questions about the content of the post). According to social exchange theory, the process of social interaction among people is a process of exchange that follows the principle of reciprocity, and the rewards of such exchange can be material or immaterial (Kankanhalli et al., 2005). As posters of novel posts are trusted and praised by other members, they will spend more time and energy participating in community discussions and contributing more personal ideas and knowledge; second, novel posters’ proactive involvement in contributing personal resources is likely to stem from the desire to experience psychological intrinsic enjoyment and pleasure again (Sethi et al., 2001). It can be inferred that posters of novel posts can actively answer questions raised by other members. Therefore, we hypothesize the following:

*H1*: Social identity is positively associated with novel posting.

*H2*: Social identity is positively associated with information support.

*H3*: Novel posting is positively associated with information support.

### Novel posting, information support, and self-identity

Researchers have argued that self-identity is formed by a process of social interaction within a particular social structure (Wojcieszak, 2021). The degree of members’ self-identity in an OKC depends on three main aspects: the degree of support an individual receives from other members, the rewards an individual receives from the role (both moral and material), and the individual’s commitment to the role and the effort that he or she puts into playing the role (Warshay et al., 1982). Novel posting and information support are two of the most important functional values of OKCs, so it is necessary for members to support those who provide such contributions. As the act of contributing benefits other members, posters of novel posts and information support are widely recognized by other members, and as a result, they develop a sense of psychological satisfaction. Both novel posting and information support require a high level of professionalism from posters and entail significant costs in terms of time and effort from posters. Novel posting and information support can be used as markers to judge contributor roles, with those who engage in more novel posting and information support being more likely to feel their contributor role (Ren et al., 2012). Based on these conclusions, we formed the following hypotheses:

*H4*: Novel posting is positively associated with self-identity as a contributor.

*H5*: Information support is positively associated with self-identity as a contributor.

### Novel posting, information support, and sense of self-worth

Individual self-esteem describes an individual’s construction of a positive self-concept, a sense of self-worth that an individual will develop based on his or her characteristics and abilities (Orth and Robins, 2022). Individual self-esteem can be divided into two levels, internal and external. The internal level is mainly expressed in the members’ desire to be a strong, competent and confident individual in OKCs; the external level refers to the individual’s desire to gain the respect, trust and praise of others in OKCs, and thus achieve a higher social status (Bleckmann et al., 2022). According to the Network Working Group perspective, an OKC is a task-and goal-oriented virtual community whose members tend to gain a sense of task accomplishment based on task contributions (Pierce and Gardner, 2004). Novel posting and information support constitute the two most important content contributions to OKCs, promoting the vitality of the community and determining the development and prosperity of the community (Moser and Deichmann, 2020). Both of these contributing behaviors can satisfy both internal and external dimensions of an individual’s self-esteem, so members can attain a sense of self-worth through their contribution behaviors, such as posting professionally or answering others’ questions actively (Yao et al., 2022). Therefore, we hypothesize the following:

*H6*: Novel posting is positively associated with sense of self-worth.

*H7*: Information support is positively associated with sense of self-worth.

### Sense of self-worth and self-identity

Based on uncertainty reduction theory, people often have a need to reduce subjective uncertainty about their personal perceptions, attitudes, emotions, behaviors, etc., and especially about the meaning of the self (Tasdemir, 2011). Reducing uncertainty about self-meaning is a core human motivation, and certainty gives individuals meaning and confidence in their existence. Weick argued that social behavioral performance can act as an observable indicator of role identity (Chiu et al., 2006). Thus, social behavior helps people perceive their particular roles. In OKCs, members’ sense of self-worth is derived from their contribution behavior (Mcallister and Bigley, 2002), with higher levels of contribution implying a greater sense of self-worth. Through in-group contribution behaviors, individuals can clarify their self-concept as contributors, which helps to guide their perceptions, emotions, and behaviors and satisfy their need to reduce subjective uncertainty (Joyce and Harwood, 2020). It can be inferred that members can perceive their role as contributors based on the sense of achievement, i.e., sense of self-worth, of contributing to OKCs (Perinelli et al., 2021). Therefore, we infer the following:

*H8*: Sense of self-worth is positively associated with self-identity as a contributor.

## Research methodology

### Research model

**Figure 1:**
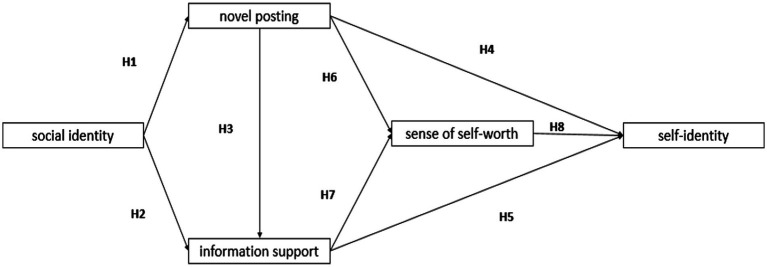
Research framework.

This research explains the process by which contributors’ self-identity is formed in the context of OKCs. We explore the relationships among social identity, sense of self-worth, information support, novel posting, and self-identity. Previous research has provided a solid theoretical basis for selecting intermediate variables and developing models; Figure 1 shows a complete path model.

### Sample and procedure

To analyze the path model, we administered an online questionnaire *via* two of the most widely used online social platforms in China (QQ and WeChat), through which people can spontaneously participate in online knowledge communities based on common interests, such as the English speaking practice group, the Python programming group, the college entrance examination group, the SPSS software online mutual aid group, and EXCEL home; thus, we ensured the generalizability of our research results. The five virtual communities (three virtual communities about college entrance exams and two virtual communities about E-Office Software Application) that participated in the data survey are typical online knowledge communities, each with a large number of members discussing relevant knowledge on a daily basis and a high level of community activity, with members in each community located across the country, and the members are spread across all age groups. Therefore, the selection of these five virtual communities for the data survey is appropriate for this study. After obtaining the consent of the relevant group owner or administrator, we posted a link to the online questionnaire to the online knowledge communities. The data collection took place over 3 months; during this time, we ultimately obtained a total of 515 usable questionnaire responses. Table 1 shows the demographic statistics for the participants.

**Table 1:** Demographic information.

Variables	Items	(%)
Gender	Male	41.2%	Female	58.8%
Education level	Lower than high school	28.7%
	Bachelor’s degree	55.7%
	Graduate degree	15.5%
Age	<20	18.1%	21–25	14.0%	26–30	14.6%	31–40	15.9%	>40	37.5%
Membership history	<1 year	29.7%	1–2 years	35.0%	>2 years	35.3%

### Measures

All measurement items in this research were drawn from measurement scales developed by previous validated research and were scored on a five-point Likert scale. To enhance the items’ validity and comprehensibility, we first administered group interviews with three professors and six senior PhD students in the program; second, we invited an English teacher from the university to translate the translated items back into English and to check them against the original English items to guarantee consistency between the initial Chinese and the English meanings; third, we recruited 15 volunteers from an online knowledge community to be measured and conducted preliminary tests of the questionnaire; and finally, we improved the items for each variable (Bock et al., 2005).

### Data analyses

We employed SPSS 22 and AMOS 22.0 for data analysis and used structural equation modeling (SEM) to test the path model and hypotheses discussed above. First, we assessed the measurement model with AMOS 22.0 software to test its reliability and validity; second, we examined the path model and hypotheses after verifying the reliability and validity of the measurement model; and third, we assessed the mediating effects in the path model. In addition, we adopted values of χ2/df ≤ 3, values of GFI, AGFI, TLI, and CFI ≥ 0.90, and an RMSEA value ≤ 0.05 as criteria for evaluating good model fit with respect to the data (Heckler, 1994).

## Results

### Measurement model

A confirmatory factor analysis (CFA) was employed to examine the reliability and validity of this measurement model. First, the test results show that the overall fitness of the measurement model meets the requirements (χ2/df = 2.041; GFI = 0.958; AGFI = 0.937; TLI = 0.976; CFI = 0.982, RMSEA = 0.045). Second, reliability was evaluated by two indicators, Cronbach’s alpha coefficient and combined reliability. Table 2 shows that the Cronbach’s α values ranged from 0.839 to 0.900 and the composite reliability values ranged from 0.842 to 0.901; all of these values were much larger than 0.7, suggesting that the measurement model had good reliability (Bagozzi and Yi, 1988). Finally, convergent validity and discriminant validity are two indicators of the validity of the measurement model. Convergent validity can be assessed using two metrics (average variance extracted and factor loading), as displayed in Table 2. All AVE values ranged from 0.641 to 0.753, significantly higher than 0.5, and Table 3 indicates that all item loading values ranged from 0.730 to 0.900, significantly greater than 0.7, thus suggesting that the measurement model had good convergent validity (Podsakoff et al., 2003; Harris and Mossholder, 1996). Discriminant validity was reviewed by contrasting the square foundation of the average variance extracted (AVE) and the shared variances. Table 4 reports that the square roots of AVE for all variables were significantly larger than the shared covariance between variables, confirming that the measurement model had good discriminatory validity.

**Table 2:** Cronbach’s alpha, composite reliability, and AVE.

Constructs	Cronbach’s α	Composite reliability	AVE
Novel posting	0.900	0.901	0.753
Self-identity	0.863	0.867	0.700
Sense of self-worth	0.850	0.850	0.654
Social identity	0.839	0.842	0.641
Information support	0.847	0.848	0.651

**Table 3:** Measurement items and standard loadings.

Variables	Measurement items	Standard loadings
**Novel posting** (Carmel et al., 2012)		
NP1	The content of my posts is new	0.841
NP2	The content of my posts is original	0.900
NP3	My posts are novel	0.859
**Self-identity** (Yun and Silk, 2011)		
SEI1	I consider myself a contributor to this community	0.851
SEI2	I recognize my role in the community as a contributor	0.872
SEI3	I consider myself a member who cares about contributing to this community	0.756
**Sense of self-worth** (Bock et al., 2005)		
SOS1	In this community, the information I provide can assist other members with resolving their issues	0.839
SOS2	This community benefits from the information I share	0.809
SOS3	I gain confidence by sharing my information to assist others within this community	0.777
**Social identity** (Swaab et al., 2007)		
SI1	I consider myself to be an important member of this community	0.730
SI2	I feel like I’m a part of this community	0.881
SI3	I feel myself to be coordinated with this community	0.784
**Information support** (Hajli, 2014)		
IS1	In this community, I usually give advice when somebody needs assistance	0.759
IS2	When somebody in this community experiences an issue, I regularly make suggestions to help that person address the issue	0.835
IS3	When somebody in this community faces challenges, I frequently help the person in question to discover the reason and give that person advice	0.825

**Table 4:** Correlations among latent constructs.

Constructs	SEI	SOS	SI	NP	IS
Self-identity (SEI)	0.837				
Sense of self-worth (SOS)	0.719	0.809			
Social identity (SI)	0.409	0.367	0.801		
Novel posting (NP)	0.717	0.715	0.358	0.868	
Information support (IS)	0.533	0.515	0.554	0.448	0.807

Data acquired through a single self-report are susceptible to common method variance (CMV; Heckler, 1994). To obviate the presence of CMV that could affect the empirical results of our data, we utilized a one-way model approach to test for common method variance. The test process required us to develop a one-factor model by linking all question items to one latent variable (Bagozzi and Yi, 1988) and then examine the path model *via* CFA. The whole fit index values performed poorly: χ2/df = 18.707; GFI = 0.645; AGFI = 0.526; TLI = 0.599; CFI = 0.656, RMSEA = 0.186, indicating that CMV had only a minimal effect on this research.

### Structural model

**Figure 2:**
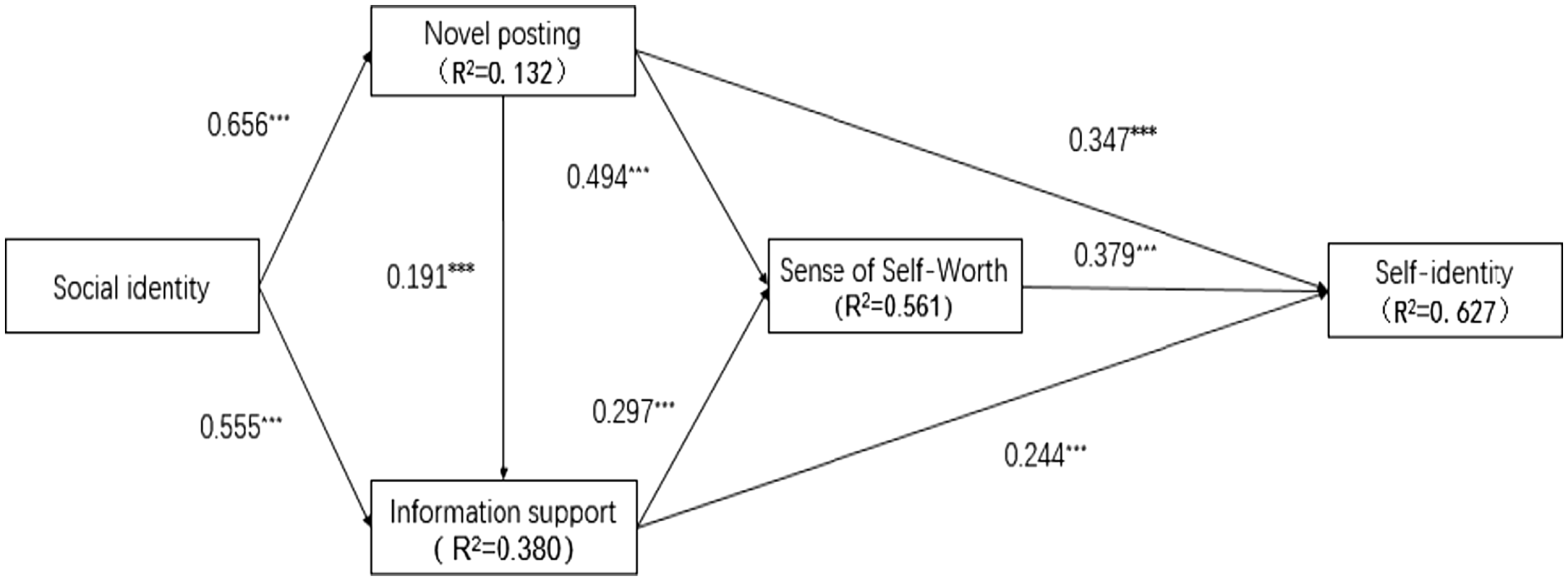
Model testing results (^***^p < 0.001).

The values of all the fitted indicators of the structural model were within acceptable limits (χ2/df = 2.017; GFI = 0.958; AGFI = 0.938; TLI = 0.977; CFI = 0.982, RMSEA = 0.044), and their values were well above their respective acceptable levels. Figure 2 displays the overall structural model with path coefficients. We found that each path coefficient was significant based on the results of structural equation analysis (*p* < 0.001), indicating that all the hypotheses are valid. The explained variances in the sense of self-worth, novel posting, information support, and self-identity were 56%, 13%, 38%, and 62%, respectively. Thus, we can conclude that the structural model had good predictive validity.

Other models were also constructed for this study, but in other models the logical relationships between variables either did not fit existing, widely accepted theory or the fit indices of new model performed poorly (e.g., construct a new model by removing path H4 with χ2/df = 3.939, RMSEA = 0.076, AGFI = 0.086. None of the values of the above indicators are within a reasonable range). In summary, the model proposed in this study not only has a solid theoretical basis but also performs well for all the overall fit indices.

### Mediation effects

**Figure 3:**
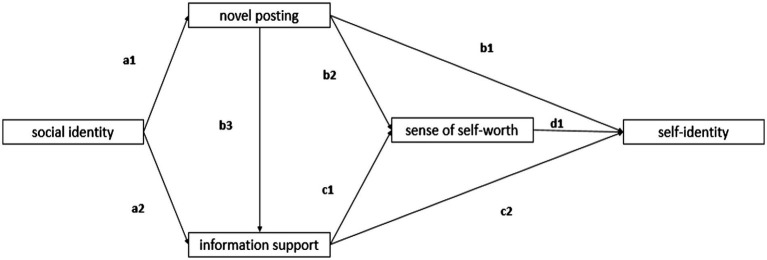
Mediation effect analysis model.

Figure 3 displays the multiple mediation model. Research has shown that bootstrapping is the best and most sensible approach for acquiring confidence limits with respect to specific indirect effects in most contexts (Preacher and Hayes, 2008). Therefore, we employed bootstrapping using AMOS 22.0 software to examine the multiple mediation model. This research investigated the indirect effects of social identity on self-identity *via* information support, novel posting and sense of self-worth.

Figure 3 shows that a1, a2, b1, b2, b3, c1, c2, and d1 represent the impacts of past factors on the resulting factors; for example, a1 indicates the impact of social identity on novel posting. Table 5 shows that there is a 95% probability that the indirect effect value of social identity on self-identity *via* novel posting ranges from 0.128 to 0.357, and the estimated effect value is 0.228. The indirect effect is significant because 0 does not appear between the lower limit value and the upper limit value. Similarly, the rest of the indirect effects of social identity on self-identity *via* sense of self-worth, information support, and novel posting are also significant.

**Table 5:** Indirect effects of social identity on self-identity *via* novel posting, information support, and sense of self-worth.

	Product of coefficients		Bootstrapping BC 95% CI	
	Point estimate	SE	Z	Lower limit	Upper limit
a1*b1	0.228	0.059	3.864	0.128	0.357
a2*b2*d1	0.123	0.035	3.514	0.063	0.201
a1*b3*c2	0.031	0.011	2.818	0.011	0.055
a1*b3*c1*d1	0.014	0.006	2.333	0.005	0.028
a2*c2	0.136	0.047	2.467	0.049	0.235
a2*c1*d1	0.062	0.023	2.894	0.024	0.114
a1*b2	0.324	0.064	5.063	0.209	0.460
a2*c1	0.165	0.044	3.750	0.082	0.256

## Discussion and implications

### Conclusion and research contributions

In this research, we constructed a path model to explore in depth the formation mechanism of self-identity as a contributor and empirically demonstrated this mechanism by collecting relevant data from participants in online knowledge communities. The R^2^ value of self-identity was 0.627, thus indicating that the model was able to explain 62.7% of the variance in self-identity. This result not only indicates that the path model can reveal the formation mechanism of self-identity as a contributor in online knowledge communities but also suggests that novel posting, information support and sense of self-worth are mediating variables that have a significant impact on the formation of self-identity.

First, self-identity as a contributor can be formed on the basis of social identity *via* the social path in OKCs. Because social identity is considered to be a core factor in the operation of OKCs (Smith et al., 2007), we introduced this key variable as the starting variable for the path analysis. The path coefficients from social identity to novel posting and information support are as high as 0.66 and 0.55 (both *p*-values ≤ 0.001), respectively. This result suggests that social identity can cause a 66% and 66% increase in novel posting and information support, respectively, allowing us to infer that social identity positively affects both novel posting and information support. It can be further inferred that the consistency of members’ attitudes and behaviors remains high in the context of OKCs and that although OKCs are online, anonymous virtual environments, the attitudes of group members continue to function as a good predictor of their behaviors. In addition, the path coefficients from novel posting and information support to self-identity are 0.35 and 0.25 (both *p*-values ≤ 0.001), respectively, which is strong evidence that both of these factors can directly improve self-identity on a behavioral level. In summary, social identity can elicit novel posting and information support, both of which further add to the development of self-identity in the context of OKCs.

Second, self-identity as a contributor is also formed *via* the psychological path in OKCs. As discussed previously, a sense of self-worth is the result of contribution (Chou et al., 2010), and a higher level of contribution entails a stronger sense of self-worth. Since novel posting and information support are the two most important contribution behaviors in OKCs, posters experience a strong sense of self-worth due to their contribution behaviors. The results of the data analysis show that the influence of sense of self-worth improves self-identity by 38% (*p*-value ≤ 0.001), implying that sense of self-worth significantly aids the establishment of self-identity. Based on these reviews, contributors’ self-identity can be cultivated *via* the mediating effect of their sense of self-worth.

Third, the two mechanisms for fostering self-identity in OKCs are not independent of each other, and there is also a very strong link between behavioral and psychological mechanisms in this context. With path coefficients of 0.35 and 0.25 from novel posting and information support to sense of self-worth, respectively, the results of the data analysis reflect that both factors can generate a sense of self-worth. This finding is consistent with previous research concerning the logical relationship between contributing behaviors and sense of self-worth. Furthermore, we find that behavioral mechanisms contribute to psychological mechanisms when they are in effect.

Fourth, the positive effects of novel posting on information support can be conducive to the sustainable development of OKCs. Social exchange theory has noted that the process of reciprocity formation is clearly social in nature, and reciprocity does not necessarily take the form of the mutual exchange of material goods (Vecchio, 2000); rather, it can also occur when people respond to friendly behavior or treatment. Posting can be viewed as a form of social exchange in OKCs; contributors gain recognition and even praise from others because of their novel posting, thus enhancing their social prestige, which can, in turn, encourage contributors to produce more novel posts. Simultaneously, group members often interact with the posters of novel posts after receiving high-quality information because there is a very convenient feedback mechanism in OKCs, and other members occasionally participate in the discussion caused by such interaction, thus making it livelier. The strong interaction among members that is triggered by the novel posting increases the activity of the community and the bonds between members and the community, which is conducive to the healthy development of OKCs.

Fifth, the dual self-running mechanism enhances the overall value of the OKCs. Social identity triggers their novel posting and information support, increasing both from zero to a positive value, while self-identity promotes novel posting and information support, increasing both from a low number to a high number, thereby improving the functional value of OKCs. The social value of OKCs thus increases, since novel posting and information support can facilitate social interaction among members. During the interactive process of participating in issue-based discussions, group members can easily find people who share their perspectives or thoughts, creating a sense of like-mindedness and psychological satisfaction and enhancing the psychological value of OKCs. It is reasonable to conclude that the value of OKCs arises primarily through the contributions of contributors, thus further reflecting the high value of contributors.

Finally, social identity in OKCs can stimulate individualized behavior that is beneficial to the group, such as novel posting. Many studies have suggested a conflict between social identity and personalization, as social identity emphasizes a convergence among members, while personalization causes individual members to behave differently from one another. Social identity can be divided into two categories: self-categorization, in which individual members self-categorize themselves into a group based on an external group characteristic, and social belonging, in which individual members develop an emotional connection to the group itself and thus a willingness to belong to it (Vecchio, 2000). Because self-categorization does not include emotion and social belonging does, social belonging is a much stronger form of identity than self-categorization. The conflict arises mainly because the specific forms of social identity employed by the relevant researchers are different. Studies arguing that personalization prevents the creation of social identity have considered personalization in the context of self-categorization, whereas studies arguing that personalization does not prevent the creation of social identity or even facilitates its formation have viewed social identity as social belonging. As mentioned previously, we can infer that personalization conflicts with self-categorization but is compatible with social belonging. After all, instead of purposefully isolating themselves from others, contributors provide high-quality professional information to OKCs as a means of capturing people’s attention and achieving a sense of self-worth.

### Theoretical implications

Our research makes theoretical contributions to the field of OKC studies.

First, previous scholars have demonstrated empirically that social identity can generate self-identity as a contributor, but due to their different research methods and purposes, they have not clarified the component variables included in this formative path and the logical relationships among them. This research develops a complete path model of the formation of self-identity on the basis of their social identity in OKCs, which shows that their novel posting, information support and sense of self-worth play mediating roles in this context. Therefore, this research fills a gap in the literature concerning the way in which the self-identity of OKCs is formed, which can, in turn, provide a reference for subsequent research pertaining to the influence of self-identity in OKCs.

Second, this research reveals the social and psychological outcomes generated by self-identity in OKCs. Previous studies have indicated that the main motivations for group members to post include the desire to express their creativity, share their industry experiences, and influence the ideas of others (Zhao et al., 2012), but these studies have failed to fully explore some of the deeper psychosocial factors underlying these motivations. Based on a model of the formation process of self-identity, we explore in depth the deeper social and psychological outcomes arising from social identity: information support, novel posting, sense of self-worth and self-identity.

Finally, this research investigates the logical relationship between social identity and individualized behavior in the context of OKCs, contributing to the development of the related field. Social identity can generate individualized behavior such as novel posting, which is beneficial to the community because it significantly enhances the functional value of the community. Novel posting promotes interaction and is therefore conducive to satisfying members’ social needs, while novel posting also triggers a sense of self-worth and is therefore conducive to satisfying members’ psychological needs. In summary, social identity and individualized behavior are not always in conflict with one another.

### Practical implications

Our research has a number of practical implications for operators managing OKCs.

First, operators should increase the social prestige of professional contributors by raising their virtual rank or pinning their posts to motivate professional contributors to post more as a form of reciprocity, thereby boosting the attractiveness of OKCs.

Second, operators must be able to identify and develop active contributors and guide them to play an active role. Active contributors of OKCs attract the most attention from group members. Their posts appear in the community almost every day, and some are even sufficiently diligent to produce dozens of posts a day. These contributors are able to attract the attention of other group members to a large extent because they are exposed to a wide range of online resources appertaining to knowledge of common interest in the community and because they are able to transmit these resources to the community in addition to actively answering questions from others. Operators can provide virtual rewards or public praise to encourage active contributors to post more often, thereby enlivening the community atmosphere and making community members more likely to remain engaged.

Third, operators can encourage members to invite high-level professionals to join the community by providing them with the latest information concerning a knowledge of interest, thereby enhancing the professionalism of the community through the professional posting of high-level members.

## Limitations and future research

This study has significant theoretical implications for research pertaining to self-identity in OKCs, but our findings inevitably face certain limitations that require further study and an expansion of research.

First, the question of the generalizability of the findings is important. Since we surveyed only five OKCs regarding college entrance exams and the E-Office Software Application and since all five of these respondents belonged to QQ groups and WeChat groups, a degree of uncertainty remains concerning whether the empirical results of this study are also applicable to other types of OKCs and other online social networking platforms.

Second, since we examined the variation in endogenous variables only partially, other influencing factors may not have been identified, such as sense of experience, social presence, resonant contagion, or immersion in the experience. These factors are common and relatively important concepts in recent research concerning OKCs, all of which critically affect the social and psychological processes that shape individuals; therefore, the roles they play in this context must be investigated and validated further in future studies.

Third, the population share of contributors in questionnaire statistics may be higher than the actual population share of contributors in online knowledge communities. Future studies may be better if they can be analyzed using objective big data based on open-source social media.

Fourth, new measurement methods need to be developed to increase the accuracy of the measurements (e.g., novel posting could be measured using unbiased rates to code discussion items posted by respondents for their relative novelty compared to OKC postings by others; information support could be measured using the relative posting frequency of survey respondents’ in response to information requests for help over a given time period, compared to the posting frequency of other community members).

Fifth, both posters and lurkers can have social identity, and it is the posters who make important contributing behaviors (novel posting and information support), but posters make up a smaller proportion of the population in OKCs. Future research may choose to analyze social identity more comprehensively and in depth from the perspective of how to motivate more lurkers with a sense of identity to contribute.

## Data availability statement

The original contributions presented in the study are included in the article/supplementary material, further inquiries can be directed to the corresponding author.

## Ethics statement

The studies involving human participants were reviewed and approved by the center for Research in Organizational Behavior, Beihang University. Written informed consent to participate in this study was provided by the participants’ legal guardian/next of kin.

## Author contributions

All authors listed have made a substantial, direct, and intellectual contribution to the work and approved it for publication. TG and ZC wrote the manuscript; ZZ and CL performed the statistics; YN and XW collected the data.

## Funding

This research was funded by Beijing Social Science Foundation grant number (16GLA007).

## Conflict of interest

The authors declare that the research was conducted in the absence of any commercial or financial relationships that could be construed as a potential conflict of interest.

## Publisher’s note

All claims expressed in this article are solely those of the authors and do not necessarily represent those of their affiliated organizations, or those of the publisher, the editors and the reviewers. Any product that may be evaluated in this article, or claim that may be made by its manufacturer, is not guaranteed or endorsed by the publisher.
